# Involvement of Carnosic Acid in the Phytotoxicity of *Rosmarinus officinalis* Leaves

**DOI:** 10.3390/toxins10120498

**Published:** 2018-11-26

**Authors:** Kwame Sarpong Appiah, Hossein Korrani Mardani, Richard Ansong Omari, Vincent Yao Eziah, John Ofosu-Anim, Siaw Onwona-Agyeman, Christiana Adukwei Amoatey, Kiyokazu Kawada, Keisuke Katsura, Yosei Oikawa, Yoshiharu Fujii

**Affiliations:** 1 United Graduate School, Tokyo University of Agriculture and Technology, 3-5-8 Saiwaicho, Fuchu, Tokyo 183-8509, Japan; ksappiah90@gmail.com (K.S.A.); hmardani26@yahoo.com (H.K.M.); talk2jafakingonline@gmail.com (R.A.O.); kkatsura@go.tuat.ac.jp (K.K.); 2 Department of International and Environmental Agriculture Science, Tokyo University of Agriculture and Technology, 3-5-8 Saiwaicho, Fuchu, Tokyo 183-8509, Japan; yosei@cc.tuat.ac.jp; 3 Department of Crop Science, University of Ghana, Legon, P.O. Box LG 44 Accra, Ghana; veziah@ug.edu.gh (V.Y.E.); camoatey@ug.edu.gh (C.A.A.); 4 School of Architecture and Science, Central University, P.O. Box 2305 Tema, Ghana; jofosuanim@gmail.com; 5 Institute of Agriculture, Tokyo University of Agriculture and Technology, 3-5-8 Saiwaicho, Fuchu, Tokyo 183-8509, Japan; agyeman@cc.tuat.ac.jp; 6 School of Life and Environmental Sciences, University of Tsukuba, Tennoudai 1-1-1, Tsukuba, Ibaraki 305-8572, Japan; kawada.kiyokazu.gu@u.tsukuba.ac.jp

**Keywords:** *Rosmarinus officinalis*, carnosic acid, allelopathy, total activity, specific activity, inhibitory, phytotoxicity

## Abstract

Weeds are rapidly developing resistance to synthetic herbicides, and this can pose a threat to the ecosystem. Exploring allelopathic species as an alternative weed control measure can help minimize the ecological threat posed by herbicide-resistant weeds. In this study, we aimed to evaluate the contribution of some polyphenols to the allelopathy of rosemary (*Rosmarinus officinalis* L.). The phytotoxic effects of rosemary (leaves, roots, inflorescences, and stems) crude extracts were tested on lettuce (*Lactuca sativa* L.). Soils incorporated with dried rosemary leaves were also tested on test plants. Reversed-phase high-performance liquid chromatography (HPLC) analysis was used to determine the content of some polyphenols (caffeic, ferulic, gallic, rosmarinic, carnosic, and chlorogenic acids) in rosemary. The specific activity and total activity of crude extracts and individual compounds were evaluated using lettuce. The crude extract of rosemary leaves showed the highest growth inhibitory effect among the rosemary organs tested. Soil amended with rosemary leaf debris reduced the dry matter and seed emergence of lettuce. Carnosic acid was the main compound detected in rosemary leaves and had a high specific activity when tested on lettuce. During the seed germination period, there was observed filter paper coloration among the test plants treated with carnosic acid (250 μg/mL). The high concentration and strong inhibitory effect of carnosic acid could explain the inhibitory activity of the rosemary leaf extract. Hence, we conclude based on the total activity estimation that carnosic acid among the other tested compounds is the major allelochemical in rosemary leaves.

## 1. Introduction

Plants produce various secondary metabolites that may have numerous biological functions when released into the environment. Some of these secondary metabolites (allelochemicals) can influence (including positive and negative effects) the growth and development of other organisms in the ecosystem through a phenomenon called allelopathy [1,2]. Some of these allelochemicals from plants are released into the environment by various plant organs including leaves, flowers, roots, fruits, and barks, among others [3]. Exudation from the roots, leachates from the leaves and other aerial parts, volatile emissions, and decomposition of plants are among the processes through which these allelochemicals are released into the environment [3,4]. Many plant species including weeds, crops, and invasive species possess this inherent potential to produce and release biologically active compounds into the environment that may have a phytotoxic effect(s) on other plants. The allelochemicals responsible for the growth inhibitory activities of some plant species were isolated and identified. l-3,4-dihydroxyphenylalanine (L-DOPA) in *Mucuna pruriens* (L.) DC. [5], cyanamide in *Vicia villosa* Roth [6], cis-cinnamoyl glucosides in *Spiraea thunbergii* Siebold ex Blume [7], rutin in *Fagopyrum esculentum* Moench [8], and angelicin in *Heracleum sosnowskyi* Manden [9], are among the identified allelochemicals from some reported allelopathic plants. The herbicidal activity of allelochemicals and the utilization of allelopathic species in sustainable weed management have been explored in various studies [10,11,12,13]. The sustainable utilization of allelochemicals and allelopathic species is especially important due to the rapid emergence of herbicide-resistant weeds [14]. Additionally, these allelochemicals among other naturally-occurring compounds are believed to decompose faster than their synthetic counterparts [13,15]. Moreover, there is a strong rationale for examining natural products to uncover potentially novel herbicide sites of action since many allelochemicals operate by mechanisms not possessed by synthetic herbicides [16,17].

Rosemary (*Rosmarinus officinalis* L.) is a shrubby herb from the Lamiaceae family that mostly grows wild in the Mediterranean. In recent times, rosemary is cultivated widely due to its myriad uses as a household spice for food flavoring in its raw or processed form. Further to this, extracts of rosemary have a long history of being used as an industrial food preservative due to its high antioxidant activity. Bioactive substances such as phenolic diterpene (carnosol, methyl carnosate, carnosic acid, and rosmanol) and phenolic acids (rosmarinic and caffeic acids) are linked with the antioxidant activity of rosemary [18,19,20]. Other studies have reported significant rosemary biological properties including hepatoprotective [21], antidepressant [22], antidiabetic [23], and antiproliferative [20]. In recent times, some medicinal plants have been reported to possess allelopathic properties, and such plants require individual attention to evaluate their contributions to sustainable weed control [24,25,26]. Although the aqueous leaf extract of rosemary was reported with potential phytotoxic activity [27], its allelopathic potentials and allelochemicals are not thoroughly studied. 

In herbicide development, compounds with high biological activity per unit weight of compound (i.e., specific activity) should be considered. Similarly, the activity of allelochemicals and allelopathic species can be assessed by their specific activity. Such compounds should have small EC_50_ values (effective concentration to induce half-maximum inhibitory action) or high specific activity [8,28]. However, the total activity of putative allelochemicals should be evaluated to determine the contribution of such compounds to the growth inhibitory effects of allelopathic species [29]. The total activity of a compound is a function of its specific activity and concentration in the plant. The total activity evaluation can be used to estimate the influence of a compound on the allelopathic effect [8,28,29,30]. Therefore, this study aimed to: (i) Check the inhibitory effects of rosemary crude extracts and some polyphenols; and (ii) estimate the contribution of the each of the tested polyphenol to the phytotoxicity of rosemary.

## 2. Results and Discussion

### 2.1. Inhibitory Effects of Crude Extracts of Rosemary Organs on Lettuce Growth

**Figure 1:**
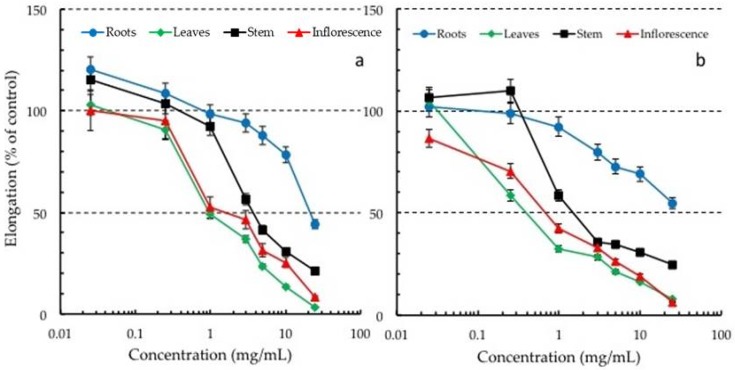
Effect of ethanol crude extract from the leaves, inflorescences, stems, and roots of rosemary plants on the radicle (**a**) and hypocotyl (**b**) growth of lettuce seedlings. The data are the mean ± standard deviation; *n* = 3.

The inhibitory effects of rosemary organs (leaves, stems, inflorescences, and roots) were tested on the radicle and hypocotyl elongation of lettuce (Figure 1). The specific activity (EC_50_) of the crude extracts from the leaves, inflorescences, stems, and roots of rosemary was in the range of 1.28–21.6 mg/mL. The application of 25 mg/mL of the crude leaf extract caused the maximum inhibition (97%) to lettuce radicle growth. However, there was no adverse effect on seed germination at this concentration. Some previous studies have shown that extracts of *Cyperus tuberosus* Bojer, *Alliaria petiolata* Cavara and Grande, and *Celastrus orbiculatus* Lam. also had no adverse effects on the seed germination of lettuce and other test plants [31,32]. 

On the contrary, some species have been reported with inhibitory effects on the germination of test plants, including lettuce, depending on the concentration of the tested plant extracts [33,34,35]. The specific activity (EC_50_) of the root (21.7 mg/mL), stem (11.1 mg/mL), and inflorescence (6.7 mg/mL) of rosemary was several times lower (about 17, 8, and 5 fold respectively) than the leaves (1.3 mg/mL). Based on the EC_50_ values, the organ order of influence on the growth inhibitory effect of rosemary on lettuce radicle growth was consequently found to be: Leaves > inflorescences > stems > roots. In general, this corresponded with the concentration of compounds found in the various organs. The content of the tested polyphenols (especially carnosic acid) was highest in the leaves compared to the inflorescences, stems, and roots as shown in Table 1. A similar trend was observed in *F. esculentum* as reported by Golisz et al. [8]. The aqueous extracts of rosemary leaves have been previously reported with some inhibitory effect on the germination and growth of *Cynodon dactylon* L. [27]. The specific activity (EC_50_) of rosemary leaves obtained in this study (1.28 mg/mL) was higher when compared to other species in existing literature. As an example, the crude extracts of *Gliricidia sepium* (Jacq.) Kunth, *Pachysandra terminalis* Siebold and Zucc., *Samanea saman* (Jacq.) Merr, and *Tamarindus indica* L showed lower inhibitory effects (EC_50_ values of 1.78, 1.92, 2.2, and 2.51 mg/mL respectively) [25]. The methanol extract of *Gliricidia sepium* leaves inhibited the radicle, and hypocotyl elongation of lettuce and the high content of coumarin explained this activity [25,36]. In other studies, *Brachiaria brizantha* (A. Rich.) Stapf with the allelochemicals (6R,9S)-3-oxo-α-ionol, (6R,9R)-3-oxo-α-ionol and 4-ketopinoresinol had an EC_50_ 2.5 mg/mL. This reported EC_50_ value for *B. brizantha* [37] was lower than that of rosemary obtained in this study. Similarly, the crude extracts of other allelopathic species like *Phragmites communis* Trin. also had lower specific activity (2.13 mg/L) [38] compared to the crude extracts of rosemary leaves.

### 2.2. The Content of Pure Compounds in Rosemary Organs

Caffeic, ferulic, gallic, rosmarinic, carnosic and chlorogenic acids were identified in the crude extracts of the tested organs of rosemary. Regardless of the plant organ, the concentration of carnosic acid was the highest among the analyzed compounds (5.3–25.3 mg g^−1^ DW), while that of ferulic acid was the least (0.01–0.8 mg g^−1^ DW) (Table 1). Similarly, Del Bano et al. [39] reported carnosic acid as the main compound in the leaves of rosemary. The results of this study showed the concentration of carnosic acid varied in the tested organs of rosemary. The compound was more abundant in the leaves, followed by the inflorescences, stems, and roots. Other studies have also shown that carnosic acid was found mainly in photosynthetic tissues (leaves, sepals, and petals), with leaves being the most abundant source (10–15 mg g^−1^ FW), followed by sepals (5–10 mg g^−1^), and petals (<2 mg g^−1^ FW) [39,40]. Rosemary cultivars with carnosic acid content of 4–10% on a weight basis of dried leaves have been developed [41]. However, the levels of carnosic acid in rosemary also depend on developmental stage, environmental conditions, among other factors [39,40] and this can influence the plant growth inhibitory activity.

Rosmarinic acid (11.9 mg g^−1^ DW) and gallic acid (1.7 mg g^−1^ DW) were the next most abundant compounds in rosemary leaves after carnosic acid. Similar levels of rosmarinic acid (8.6–15.3 mg g^−1^ DW) and caffeic acid (0.012–0.2 mg g^−1^ DW) have been detected in rosemary [20,40,42]. Caffeic acid (0.01–0.45 mg g^−1^ DW), chlorogenic acid (0.01–0.9 mg g^−1^ DW) and ferulic acid (0.01–0.8 mg g^−1^ DW) were detected at lower concentrations compared to carnosic acid and rosmarinic acid in all of the rosemary organs tested. The concentration of caffeic acid was about ≈56-fold lower than that of carnosic acid. Low concentrations of chlorogenic acid (1.07 µg g^−1^), ferulic acid (2.0 µg g^−1^), and caffeic acid (12.6 µg g^−1^) have been reported in the leaves of rosemary [43].

### 2.3. Plant Growth Inhibitory Effects of Pure Compounds Present in Rosemary

**Figure 2:**
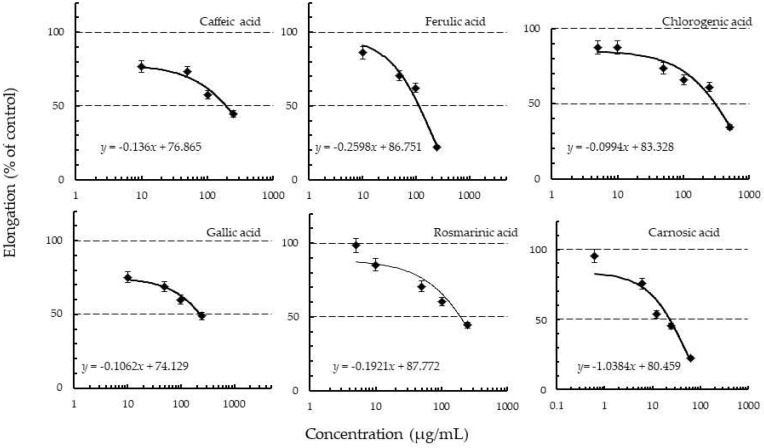
The effects of pure compounds on the radicle growth of lettuce seedlings. The data are the mean ± standard deviation of three replications.

Caffeic, ferulic, chlorogenic, gallic, rosmarinic, and carnosic acid were tested for their plant growth inhibitory effect on lettuce. The examined polyphenols showed various degrees of inhibitions on lettuce radicle and hypocotyl elongation (Figure 2). Except for rosmarinic and carnosic acids, there are reports of plant growth inhibitory effects of all the bioactive compounds assayed in this study [44,45,46]. In general, lettuce radicle elongation was inhibited more than the hypocotyl elongation for all the compounds tested in this study. For example, the EC_50_ on lettuce hypocotyl elongation was 330 µg/mL for caffeic acid and 207 µg/mL for ferulic acid, whereas that on the radicle was 197 and 141 µg/mL respectively. Yukiko et al. [45] reported that some of the polyphenols tested in this study (Chlorogenic, ferulic, caffeic, and gallic acids) also inhibited the radicle growth of *Betula platyphylla* Sukatchev var. *japonica* and *Lactuca sativa* L more than the hypocotyl growth. Another study by Fujii et al. [5] also indicated that L-DOPA had either no or very little influence on the hypocotyl growth of lettuce. Chon and Kim [47] however reported that ferulic and caffeic acids caused slight stimulation to the hypocotyl growth of alfalfa. Carnosic acid showed the highest inhibitory effect on lettuce radicle, followed by ferulic and rosmarinic acids, with specific activity values (EC_50_) of 29, 141, and 196 µg/mL respectively (Figure 2). Chlorogenic acid had the lowest inhibitory activity with an EC_50_ value of 335 µg/mL. However, gallic acid and caffeic acid showed a slight inhibitory effect on lettuce radicle elongation. 

### 2.4. Estimation of Contributions of the Various Compounds to Rosemary Growth Inhibitory Activity 

**Figure 3:**
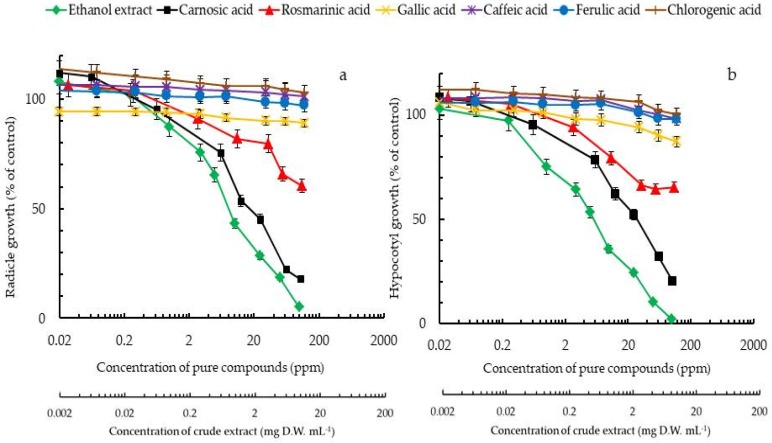
Inhibition activity of the radicle (**a**) and hypocotyl (**b**) growth of lettuce seedlings caused by the ethanol extract of rosemary leaves and by pure chemicals estimated to be present in the ethanol extract. The data are mean ± standard deviation; *n* = 3.

The inhibitory effects of the individual polyphenols and that of the crude extracts of rosemary leaves on the radicle and hypocotyl elongation of lettuce seedlings were compared to find the compound with the most significant contribution to rosemary allelopathy (i.e., total activity). This approach of estimating the most important plant growth inhibitor based on concentration and inhibitory effect (specific activity or EC_50_) of the said compound have previously been used to identify some important allelochemicals including cyanamide, juglone, angelicin, L-DOPA, durantanins, rutin umbelliferone, among others [7,8,9,26,30]. Since the inhibitory effect on lettuce by rosemary leaves was the highest among the other parts of the plant (Figure 2), we present the total activity estimation based on the leaves. As an example, the content of carnosic acid in 1.0 mg/mL rosemary leaves was determined to be 25 µg/mL. The lettuce radicle elongation as a result of the 25 µg/mL carnosic acid (estimated amount in the leaf extract) was determined to be 45.3%. Therefore, the equivalent elongation that indicates the inhibition effect of the estimated carnosic acid in the 1.0 mg/mL of rosemary extract was obtained to be 45.3%. Following the same method of calculation, the contribution of the other compounds were estimated. The inhibitory effect of carnosic acid (based on the concentration obtained in rosemary leaf extract) and whole crude extracts has more analogous volumes than the other polyphenols. The inhibitory effect of *R. officinalis* leaf extract on both the radicle and hypocotyl growth of lettuce was completely explained by carnosic acid in the extract (Figure 3). Based on this estimation, carnosic acid is the compound most responsible for the inhibitory activity of the crude extracts rosemary leaves. The high concentration of carnosic acid in rosemary organs coupled with high specific activity further show the importance of the compound in the plant growth inhibition observed. 

Carnosic acid was identified in only a few plant species, among which all belong to the Lamiaceae family. Presently, *R. officinalis* is considered to be the most abundant source of carnosic acid [44,48]. Carnosic acid and its derivative carnosol, have been suggested to be the major contributors to the antioxidant properties of rosemary extract [18,49,50].

### 2.5. Growth Inhibition and Changes in Filter Paper Coloration by Carnosic Acid 

**Figure 4:**
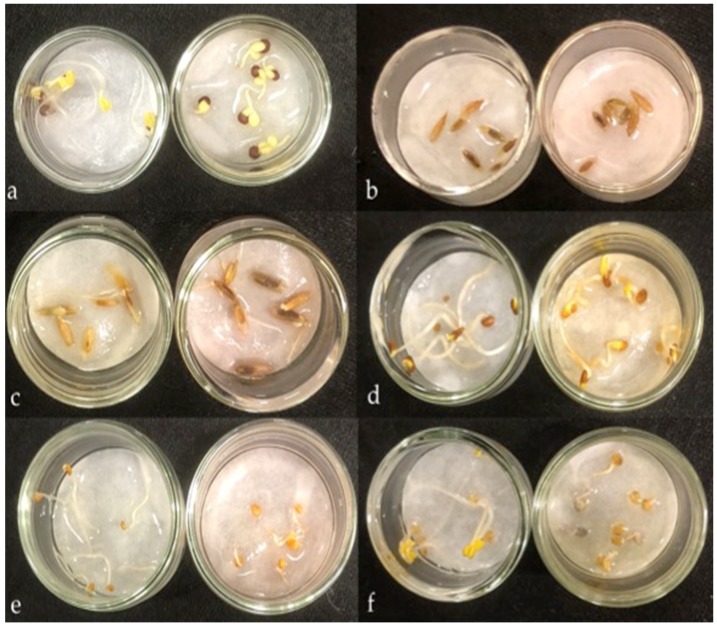
Comparison of filter paper coloration in different plants after a 3-day incubation with carnosic acid (250 µg/mL) solution. (**a**) Pak choi, (**b**) Kentucky bluegrass, (**c**) Perennial Ryegrass, (**d**) Alfalfa, (**e**) White clover, and (**f**) Arugula. Left side: Control, Right side: Carnosic acid.

The growth inhibition of radicle and hypocotyl of test plants and the changes in coloration of filter paper for different species treated with carnosic acid during the germination period are shown in Table 2. Generally, the inhibition of radicle elongation by carnosic acid was higher than that of the hypocotyl for all the test species. Additionally, some of the radicle tips of test plants showed dark coloration, curvature, or spiral growth. Carnosic acid inhibited the radicle growths of tested species from the Poaceae and Brassicaceae families more than the species from other plant families. The radicle elongation inhibition for all the species tested was more 50% except the tested species from the Apiaceae family (*Foeniculum vulgare* Mill. and *Daucus carota* L.), suggesting that carnosic acid could be less effective against plants of the Apiaceae family. Although some plants in the Apiaceae family including carrot, celery, fennel, and parsley have been reported with different response to herbicides, the mechanism behind the response is still unknown. Smith [51] reported that the post emergence application of prometryn was very safe to carrot, while linuron reduced the growth of fennel. Conversely, the inhibitory effects of carnosic acid on the hypocotyl growths of test plants were more variable (Table 2). The inhibition of hypocotyl elongation of four species (*Celosia cristata* L., *Foeniculum vulgare* Mill., *Daucus carota* L., and *Festuca pratensis* Huds.) was below 50% of the control. Some of the filter papers showed coloration after three days of incubation of carnosic acid treated seeds (Figure 4). The species from the families of Amaranthaceae (*Gomphrena globosa* L. and *Celosia cristata* L.) and Lamiaceae (*Ocimum basilicum* L. and *Rosmarinus officinalis* L.) did not cause any coloration of filter paper during the seed germination period. However, tested species of Poaceae (except *Phleum pratense* L.), Fabaceae (except *Medicago sativa* L. which showed yellowish), and Apiaceae showed reddish coloration, and Brassicaceae (excluding *Brassica rapa* subsp. Rapa L.) showed grey coloration. About the Asteraceae family, lettuce induced grey coloration of the filter paper, but crown daisy did not induce any color change. In the case of *V. villosa*, both the control and the carnosic acid treatment caused reddish coloration. 

Three types of coloration responses including black and grey (Poaceae and Compositae), no change (Fabaceae, Brassicaceae, and Cucurbitaceae) and an obstructed-circle around the seeds with black coloration on the outer side of the circle (Hydrophyllaceae) were reported when L-DOPA solution was applied during seed germination [52]. Radicle growth inhibition of all test plants that resulted in coloration of filter paper (reddish, yellowish, or grey) was minimal compared to no coloration except for *Lolium multiflorum* Lam, *Eruca sativa* Mill, and *Lactuca sativa* L. Nonetheless, the relationship between the changes in filter paper coloration and the inhibition of radicle growth in this study is still unknown. However, the filter paper coloration appears to be a response by some of the species to protect the cell from the phytotoxicity of carnosic acid. Carnosic acid is considered relatively unstable, particularly in a solvent and its degradation reaction can be induced by air oxidation [50,53]. Two quinone derivatives; *o*-quinone and hydroxy *p*-quinone were produced by carnosic acid during the mechanism of oxidation and conversion pathway [48,54]. However, the extreme oxidation of phenolic compounds often gives colored products [55]. The filter paper coloration is likely due to the oxidized compound produced during the germination period. Nishihara et al. [52] reported a similar observation from the L-DOPA, an allelochemical from *M. pruriens*. It is thought that some organisms metabolize L-DOPA to dopamine and a melanin-like compound that are less inhibitory than L-DOPA [56,57].

### 2.6. Effects of Increasing Seed Number of Test Plants on Phytotoxicity of Carnosic Acid

**Figure 5:**
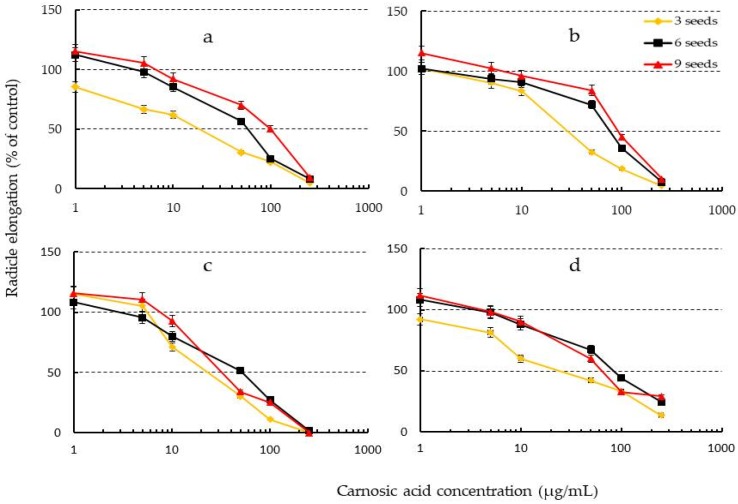
Effects of seed number variation and carnosic acid concentration on radicle growth of (**a**) Pak choi, (**b**) Lettuce, (**c**) Timothy, and (**d**) White clover.

We hypothesized that carnosic acid might be easily decomposed by enzymes in the seeds or radicles when the number of seeds increased. Based on this assumption, the number of seeds from four different plant families was altered, and the inhibition effect of carnosic acid was tested. The test species were timothy (*Phleum pratense* L., Poaceae), lettuce (*Lactuca sativa* L., Asteraceae), white clover (*Trifolium repens* L., Fabaceae), and Pak choi (*Brassica rapa* subsp. *Chinensis* (L.) Hanelt, Brassicaceae). The half-maximal inhibition (EC_50_) of all test species caused by carnosic acid was achieved even by increasing the number of seeds. In the case of Pak choi, the EC_50_ values for the different seed numbers were 28.2, 96.5, and 125 µg/mL respectively for 3, 6, and 9 seeds. The resulting inhibitory effect of carnosic acid reduced when the number of seeds of each test plant was increased (Figure 5). Nishihara et al. [52] also reported that the inhibition effects of L-DOPA reduced when the number of seeds of test plants including perennial ryegrass, white clover, lettuce and bluebell increased.

### 2.7. Effect of Soil-Incorporated with Rosemary Leaf Debris on the Bioassay Species

**Figure 6:**
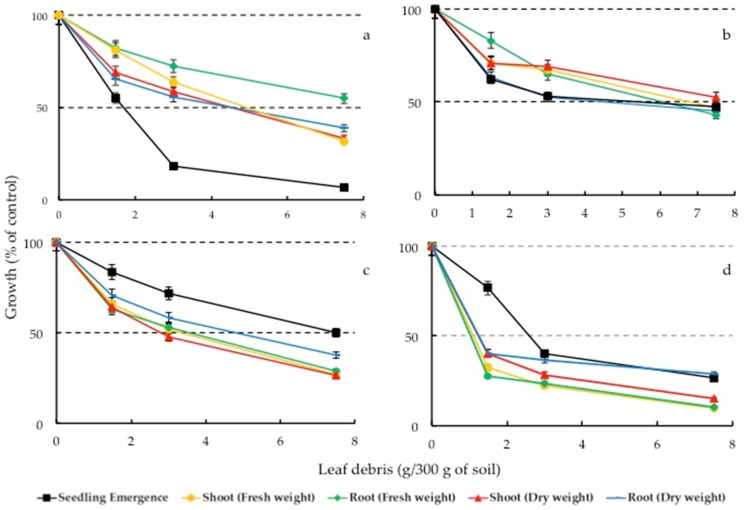
Effect of soil incorporated leaf debris of *Rosmarinus officinalis* on the growth of *Lactuca sativa* (**a**), *Lolium multiflorum* (**b**), *Trifolium repens* (**c**), and *Phleum pratense* (**d**).

The dried leaves of rosemary were incorporated into the soil to evaluate the effect on test plants. In general, the shoot (fresh and dry weights) of all test plants was inhibited more than the root (fresh and dry weights) for all the test plants as shown in Figure 6. On the contrary, Cheema et al. [58] reported that the residues of sorghum and *Brassica campestris* L. affected the root dry weight of *Trianthema portulacastrum* L. more than the shoot dry weight. The incorporation of rosemary leaf debris affected the growth of lettuce at all application rates. With the increased application rate, the emergence of lettuce declined tremendously. At 7.5 g leaf debris per 300 g soil, there was up to a 93.3% reduction in lettuce seed emergence compared to the control (Figure 6a). At a concentration of 7.5 g per 300 g of soil, the inhibitions of dry and fresh weights of lettuce root were 61% and 45% respectively. The emergence of Italian ryegrass was however not markedly affected at any of the applied rates. The maximum inhibition of 53% on Italian ryegrass emergence occurred at the highest application rate of 7.5 g per 300 g of soil. Similarly, the inhibition of root and shoot growth of Italian ryegrass was not remarkable as the maximum inhibition of 57% and 53% were respectively observed in the root and shoot fresh weight at same application rate. Likewise, the emergence of white clover (*Trifolium repens*) was not inhibited beyond 50%, even at the highest incorporation rate (Figure 6c). However, the root (fresh and dry) and shoot (fresh and dry) were inhibited above 50%. The maximum inhibition (73%) was observed in the shoot dry weight 7.5 g per 300 g soil. None of the application rates of rosemary incorporation resulted in stimulation of any of the growth parameters contrary to the stimulatory effect of *Pennisetum purpureum* on *Eleusine indica* (L.) Gaertn reported by Ismail et al. [59]. *Phleum pratense* (Timothy) showed the highest sensitivity to the growth inhibitory substances released by rosemary leaves. All the growth parameters excluding seedling emergence were inhibited even at the minimum incorporation rate of 0.5%. This inhibition trend indicates that the phytotoxicity of the active inhibitory substance(s) in rosemary leaves could be post-emergent. The observed growth parameters of Timothy and lettuce showed a similar inhibition trend when treated with soil incorporated with *Ophiopogon japonicas* Wall [60]. The phenomenon of seedling growth as affected by soils amended with the debris of allelopathic plants have been reported in other studies [61].

## 3. Conclusions

Our results showed that the leaves of *Rosmarinus officinalis* (both extracts and debris) are highly allelopathic. The crude extract of rosemary leaves inhibited the lettuce radicle elongation by 50% at 1.3 mg mL^−1^. Carnosic acid was estimated to be responsible for the plant growth inhibitory activity of rosemary leaves. Carnosic acid inhibited several species from a broad category of plant families, and there were various coloration responses during germination. Although there was a filter paper coloration response to carnosic acid during the germination period, the mechanism(s) underlying this filter paper coloration is unknown. This is the first report of carnosic acid as a plant growth inhibitor from *R. officinalis*. Nonetheless, the exact mechanism(s) of action of a carnosic acid as a plant growth inhibitor needs to be explored further.

## 4. Materials and Methods 

### 4.1. Chemicals and Test Plants for Bioassay

**Figure 7:**
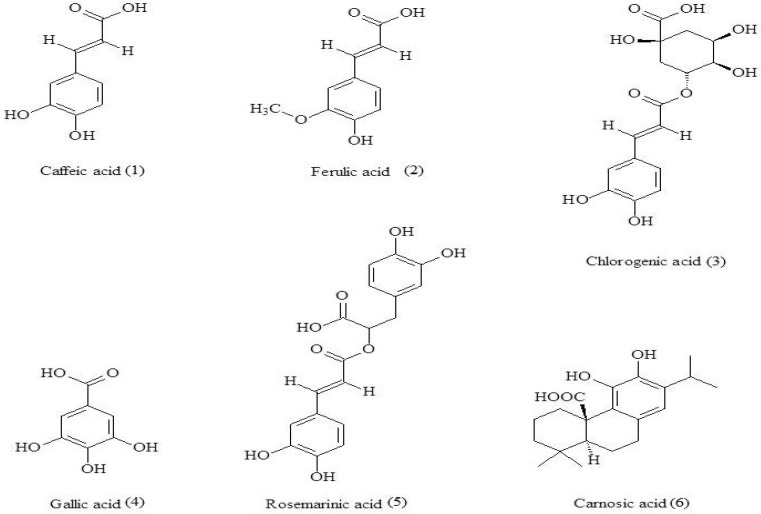
Bioactive compounds used in this study.

Caffeic acid was purchased from Nacalai Tesque Inc. (Kyoto, Japan). Ferulic acid and carnosic acid were purchased from Tokyo Chemical Industry, (TCI, Tokyo, Japan). Chlorogenic acid was purchased from MP Biomedicals, LLC (Santa Ana, CA, USA) Gallic acid purchased from Wako Pure Chemical Corporation (Osaka, Japan) and rosmarinic acid was purchased from Sigma-Aldrich (Suisse, Switzerland). All compounds (Figure 7) were reagent grade and were used without further purification. Seeds of 21 plants from seven different plant families were purchased from local seed companies in Japan and used as test plants. The seeds belonged to the following plant families; Amaranthaceae (*Gomphrena globosa* and *Celosia cristata*), Apiaceae (*Daucus carota* and *Foeniculum vulgare*), Asteraceae (*Lactuca sativa* and *Glebionis coronaria*), Brassicaceae (*Glebionis coronaria, Brassica rapa* var. *perviridis, Brassica rapa* subsp. *Chinensis, Raphanus raphanistrum, Brassica rapa subsp. Rapa,* and *Eruca sativa*), Fabaceae (*Trifolium repens, Vicia villosa,* and *Medicago sativa*), Lamiaceae (*Ocimum basilicum,* and *Rosmarinus officinalis*), and Poaceae (*Festuca pratensis, Lolium perenne, Lolium multiflorum, Poa pratensis,* and *Phleum pratense*).

### 4.2. Selection of Rosemary Samples and Extraction Procedure

*Rosmarinus officinalis* leaves showed potential plant growth inhibitory effect on lettuce growth elongation in the initial screening for potential allelopathic species among medicinal plants (Table S1). Subsequently, rosemary seeds (rosemary 116/1) were purchased from Shibata Gardening Co. (Saitama, Japan) and grown in pots in 2016. The crude extracts were obtained from the air-dried rosemary plant samples (leaves, inflorescences, stems, and roots), which were finely ground and extracted twice with 80% ethanol for 48 h at room temperature. The solution was sonicated (10 min), filtered (No. 1 filter paper, Advantec Toyo Roshi Kaisha, Tokyo, Japan), centrifuged using Hitachi himac CR22N (6000 rpm, 10 min), and the supernatants were collected. The residue was re-extracted following the same procedure as above and the supernatants were combined and used as a working solution. 

### 4.3. Inhibitory Effects of Rosemary Crude Extract and Test Compounds

The specific activity of crude extracts and the tested compounds (Figure 7) was evaluated using lettuce as a test plant [8]. The crude extracts were obtained from the leaves, stems, roots, and inflorescences of rosemary as described in the extraction procedure. The following concentrations of the crude extracts were tested; 0.025, 0.25, 1.0, 3.0, 5.0, 10, and 25 mg/mL DW. Filter paper (27 mm ø, Toyo Roshi Kaisha, Ltd., Tokyo, Japan) was placed in a glass Petri dish (27 mm ø). A total of 0.7 mL of test solution was added to the filter paper and dried completely in vacuo. Five pre-germinated seedlings were placed on the filter paper after adding 0.7 mL of 0.05% Dimethyl sulfoxide (DMSO) and incubated (CN-25C, Mitsubishi Elec., Tokyo, Japan) for 52 h at 22 °C in dark conditions. The inhibitory activity bioassays using rosmarinic acid, ferulic acid, caffeic acid, chlorogenic acid, gallic acid, and carnosic acid were done in the same conditions described above. The contribution of each polyphenol to the allelopathy of rosemary crude extract was estimated based on the inhibition and concentration of the compounds in the crude extract (i.e., total activity). The control treatments were set up with no crude extract or compound but only 0.05% DMSO. Three replications were set for each treatment. The radicle and hypocotyl lengths were measured after the incubation period and the elongation percentage was calculated using equation (1) below modified from Chandra et al. [62]:

(1) $$\;Elongation\%=\frac{X}{Y}\times 100\;$$

where, *X* = Treated (crude extract or pure compound) mean radicle/hypocotyl length and *Y* = Control mean radicle/hypocotyl length. 

### 4.4. HPLC Analysis of Rosemary Organs 

A total of 50 mg of ground rosemary samples (leaves, stems, inflorescences, and roots) were accurately weighed into a 50 mL tube and extracted as shown in the extraction procedure. An aliquot of the extract after centrifugation was filtered through a 0.2 μm syringe filter before injection (10 µL). HPLC analysis was carried out using an LC-20AD liquid chromatograph (Shimadzu, Japan). An Inertsil ODS 2 column (250 × 4.6 mm, 5 μm particles, GL Sciences Inc, Tokyo, Japan) was used. Mobile phases A and B were water with 0.1% formic acid and acetonitrile, respectively. The column temperature was kept at 30 °C, and the flow rate of the mobile phase was set at 0.5 mL min^−1^. The following multi-step gradient with different proportion of mobile phase B was applied: 0 min, 20% B; 10 min, 40% B; 15 min, 90% B and maintained for 5 min. The initial conditions were maintained for 5 min. The analysis was monitored by using SPD-M20A detector at 280 nm. The quantification was obtained by comparing the peak areas of the target commercial compounds with the abundance of these compounds in corresponding standards used in the calibration curve. All chemical analyses were done in three replicates. 

### 4.5. Growth Inhibition and Filter Paper Coloration Changes Induced by Carnosic Acid

In this inhibitory activity bioassay, twenty-one test plants from seven different plant families were used in germination tests. Seeds of each of the test plants were placed in 27 mm diameter Petri dishes that contained 0.7 mL of either distilled water (control) or carnosic acid solution (250 µg/mL) on filter paper. Each dish was placed on a soaked double Kimtowel (Kimberly-Clark Co., Tokyo, Japan) to maintain moisture in the dishes and finally kept in an incubator (CN-25C, Mitsubishi Elec., Tokyo, Japan) under dark conditions for three days at 22 °C. The changes in the coloration of the filter paper in each dish were monitored and photographed. The radicle and hypocotyl lengths of each of the test plants were measured on day three. The radicle and hypocotyl growth were expressed as a percentage of the water control (using Equation (1)).

### 4.6. Effects on Growth Inhibition Activity of Carnosic Acid by Varied Seed Number of Test Plants

Based on the earlier growth inhibition bioassay experiment, four test plants from four different plant families were selected to investigate 50% inhibition (EC_50_) of radicle growth. Various combinations of carnosic acid concentrations (1, 5, 10, 50, 100, and 250 µg/mL) and numbers of seeds (3, 6, and 9) from each plant species were investigated in 27 mm Petri dishes which contained either 0.7 mL of carnosic acid solution or distilled water. The experimental procedure was the same as phytotoxic bioassay test described in Section 4.3. The radicle and hypocotyl lengths were measured on day three and expressed as a percentage of the control.

### 4.7. Effect of Rosemary Leaf Debris Incorporated Soil on the Bioassay Species 

This study was conducted to determine whether leaf debris of rosemary would have any effect on the growth of test plants under greenhouse conditions. Pre-weighed 300 g of soil (Kumiai Nippi, Tokyo, Japan) were autoclaved and mixed with 0, 1.5, 3.0, and 7.5 g of rosemary dried leaf debris. Hence, the treatments were expressed as 0, 0.5%, 1.5%, and 2.5% (*w*/*w*) based on the soil dry weight. These are consistent with the incorporation rate used for the allelopathic studies of other plant species [59,60]. The treatment mixture was thoroughly mixed and transferred into rectangular plastic pots. Twenty seeds of *Lactuca sativa*, *Lolium multiflorum*, *Trifolium repens*, and *Phleum pratense* were sown in separate boxes. There were four replications of each concentration for each of the bioassay species. The pots were placed in the greenhouse and watered daily. The average temperature during the growth of the plants was 26 °C and 19 °C respectively for day and night. Emergence was recorded after four days of planting and after the all seeds of the control had germinated (approximately five days after planting). Plants were harvested four weeks after planting. The roots were carefully separated from the soil by gently washing the soil. Seedling emergence and root and shoot weights (fresh and dry) were measured; data were converted to a percentage of the control.

### 4.8. Statistical Analysis

The results obtained were statistically analyzed by one factor of the ANOVA test. Values of *p* < 0.05 when different to the control were considered statistically significant.

## Figures and Tables

**Table 1:** Concentration of biologically active compounds in the tested organs of rosemary.

Compound	Total Amount in Each Organs (mg g^−1^ DW)				Concentration of Compounds in Examined Organs of Rosemary (mg g^−1^ DW)
	Leaves	Inflorescences	Stems	Roots	
**Caffeic acid**	0.45 ± 0.01	0.02 ± 0.01	0.01 ± 0.00	ND	0.48a
**Ferulic acid**	0.81 ± 0.03	0.01 ± 0.00	0.02 ± 0.01	0.01 ± 0.00	0.85a
**Gallic acid**	1.72 ± 0.31	0.02 ± 0.00	0.01 ± 0.00	ND	1.72a
**Carnosic acid**	25.3 ± 3.21	12.5 ± 1.36	8.31 ± 0.96	5.32 ± 0.85	51.4b
**Chlorogenic acid**	0.92 ± 0.12	0.01 ± 0.00	ND	0.02 ± 0.00	0.95a
**Rosmarinic acid**	11.9 ± 1.21	4.52 ± 0.65	3.21 ± 0.31	3.51 ± 0.65	23.1c
**Amount in Organ**	41.1b	17.1a	11.6a	8.86a	-

Values presented are mean ± standard deviation of triplicates. The numbers followed by the same letters are not significantly different (*p* < 0.05). All compounds were quantified by comparison with corresponding commercial standards. ND; Not Detected.

**Table 2:** Radicle and hypocotyl growth and coloration changes of filter paper in germination tests with 250 µg/mL carnosic acid treatment.

Family	Common Name	Scientific Name	Elongation (% of Control)		Paper Coloration
			Radicle	Hypocotyl	
Amaranthaceae	Globe amaranth	*Gomphrena globosa* L.	23.5 ± 2.05	45.3 ± 4.33	No change
	Common cockscomb	*Celosia cristata* L.	26.4 ± 2.13	65.0 ± 10.3	No change
Apiaceae	Fennel	*Foeniculum vulgare* Mill.	67.6 ± 3.15	83.8 ± 12.3	Reddish
	Carrot	*Daucus carota* L.	74.5 ± 4.35	86.3 ± 13.5	Reddish
Asteraceae	Lettuce	*Lactuca sativa* L.	6.0 ± 1.05	13.2 ± 0.89	Grey
	Crown daisy	*Glebionis coronaria* (L). Cass. Ex. Spach	28.7 ± 2.19	32.7 ± 2.56	No change
Brassicaceae	Komatsuna	*Brassica rapa* var. *perviridis* L.H. Bailey	10.0 ±1.56	24.8 ± 2.01	Grey
	Pak choi	*Brassica rapa* subsp. *Chinensis* (L.) Hanelt	9.1 ± 1.15	37.9 ± 2.65	Grey
	Radish	*Raphanus raphanistrum* subsp. *Sativus* (L.) Domin	15.4 ± 1.86	28.4 ± 2.89	Grey
	Turnip	*Brassica rapa* subsp. *Rapa* L.	7.7 ± 0.11	31.9 ± 2.66	No change
	Arugula	*Eruca sativa* Mill.	7.3 ± 0.15	21.7 ± 1.95	Grey
Fabaceae	White clover	*Trifolium repens* L.	25.6 ± 2.25	21.2 ± 1.80	Reddish
	Hairy vetch	*Vicia villosa* Roth	28.9 ± 2.6	40.9 ± 3.10	Reddish
	Alfalfa	*Medicago sativa* L.	31.7 ± 2.65	34.7 ± 5.33	Yellowish
Lamiaceae	Basil	*Ocimum basilicum* L.	26.9 ± 1.56	35.4 ± 4.22	No change
	Rosemary	*Rosmarinus officinalis* L.	22.1 ± 1.31	28.3 ± 3.33	No change
Poaceae	Meadow fescue	*Festuca pratensis* Huds.	41.2 ± 3.01	59.5 ± 7.56	Reddish
	Perennial ryegrass	*Lolium perenne* L.	26.6 ± 2.66	38.9 ± 6.44	Reddish
	Italian ryegrass	*Lolium multiflorum* Lam.	6.2 ± 0.01	49.1 ± 7.56	Reddish
	Kentucky bluegrass	*Poa pratensis* L.	12.4 ± 1.23	37.8 ± 5.66	Reddish
	Timothy grass	*Phleum pratense* L.	3.8 ± 0.02	7.0 ± 0.89	No change

Data are mean of three replications ± Standard Deviation (SD).

## Supplementary Materials

The following are available online at http://www.mdpi.com/2072-6651/10/12/498/s1, Table S1: Allelopathic activities of medicinal plants collected from southern Ghana using the Sandwich method.
