# Supplementary material for: Involvement of Carnosic Acid in the Phytotoxicity of Rosmarinus officinalis Leaves
Source: Toxins (Basel). 2018 Nov 26;10(12):498. doi: 10.3390/toxins10120498 (PMC6316382; doi:10.3390/toxins10120498)
Supplement: Supplementary file 1 [file toxins-10-00498-s001.pdf]

Toxins **2018**, *10*, 498; doi: 10.3390/toxins10120498

Supplementary Materials: Involvement of Carnosic Acid in the Phytotoxicity of *Rosmarinus officinalis* Leaves

Kwame Sarpong Appiah, Hossein Korrani Mardani, Richard Ansong Omari, Vincent Yao Eziah, John Ofosu-Anim, Siaw Onwona-Agyeman, Christiana Adukwei Amoatey, Kiyokazu Kawada, Keisuke Katsura, Yosei Oikawa and Yoshiharu Fujii

Table S1. Allelopathic activities of medicinal plants collected from southern Ghana using the Sandwich method.

No. of Plants	Family	Part Used	Scientific Names	Growth Elongation (% of Control)				Criteria
				10 mg		50 mg		
				Radicle	Hypocotyl	Radicle	Hypocotyl	
1	Euphorbiaceae	Leaves	*Bridelia micrantha* Baill.	2.7	2.0	1.7	2.1	****
2	Myrtaceae	Leaves	*Syzygium malaccense* (L.) Merr. & L.M.Perry	3.9	6.3	2.5	8.0	****
3	Lamiaceae	Leaves	*Rosmarinus officinalis* L.	4.3	5.6	2.1	2.0	****
4	Apocynaceae	Seeds	*Voacanga africana* Stapf ex Scott Elliot	9.2	21.2	3.2	8.7	***
5	Caesalpiniaceae	Leaves	*Delonix regia* (Bojer) Raf.	9.7	35.8	6.1	27.4	***
6	Fabaceae	Leaves	*Pterocarpus santalinoides* L'Hér. ex DC.	12.2	30.8	5.6	19.0	**
7	Fabaceae	Leaves	*Dialium guineense* Willd.	13.4	32.7	3.5	9.5	**
8	Fabaceae	Leaves	*Piliostigma thonningii* (Schumach.) Milne-Redh.	13.6	54.2	5.4	18.8	**
9	Caricaceae	Leaves	*Carica papaya* L.	14.3	37.6	8.4	27.3	**
10	Fabaceae	Leaves	*Tephrosia purpurea* (L.) Pers.	15.1	61.2	12.7	31.6	**
11	Malvaceae	Leaves	*Malvastrum coromandelianum* (L.) Garcke	18.9	76.7	15.4	50.5	*
12	Solanaceae	Leaves	*Datura metel* L.	19.2	61.6	7.5	33.6	*
13	Sapotaceae	Leaves	*Chrysophyllum albidum* G.Don	19.5	50.4	14.7	29.4	*
14	Astaraceae	Leaves	*Chromolaena odorata* (L.) R.M King & H.Rob.	21.1	94.2	20.8	72.6	
15	Cucurbitaceae	Leaves	*Momordica charantia* L.	21.3	69.9	11.0	46.5	
16	Apocynaceae	Stem bark	*Saba florida* (Benth.) Bullock	22.9	65.0	12.0	44.3	
17	Fabaceae	Leaves	*Cassia biflora* L.	23.4	48.9	7.2	16.1	
18	Sapindaceae	Leaves	*Blighia sapida* K. D. Koenig	24.3	47.2	12.2	22.2	
19	Fabaceae	Leaves	*Milletia thonningii* (Schum. & Thonn.) Baker	24.3	57.3	14.4	43.9	
20	Euphorbiaceae	Root bark	*Bridelia micrantha* Baill.	25.0	73.0	10.0	44.0	
21	Rutaceae	Leaves	*Clausena anisata* Hook.f., De Wild. & Staner	26.1	36.5	15.4	31.2	
22	Fabaceae	Leaves	*Cassia siamea* Lam.	26.2	52.1	5.2	11.4	
23	Fabaceae	Leaves	*Leucaena leucocephala* (Lam.) de Wit	26.5	72.2	11.6	41.7	

Table S1. continued.

No. of Plants	Family	Part Used	Scientific Names	Growth Elongation (% of Control)				Criteria
				10 mg		50 mg		
				Radicle	Hypocotyl	Radicle	Hypocotyl	
24	Meliaceae	Leaves	*Entandrophragma cylindricum* Sprague	27.6	92.4	15.3	56.8	
25	Rubiaceae	Leaves	*Psydrax subcordata* (DC.) Bridson	27.7	40.0	17.2	47.9	
26	Anacardiaceae	Leaves	*Anacardium occidentale* L.	27.8	80.0	16.1	61.7	
27	Apocynaceae	Leaves	*Mondia whiteii* (Hook.f.) Skeels	28.1	44.0	13.2	26.0	
28	Moraceae	Leaves	*Treculia africana* Decne. ex Trécul	28.2	80.0	20.4	57.8	
29	Euphorbiaceae	Stem bark	*Bridelia micrantha* Baill.	28.2	87.8	15.4	52.3	
30	Fabaceae	Leaves	*Parkia biglobosa*	29.8	62.7	14.9	35.7	
31	Moraceae	Leaves	*Ficus exasperata* Roxb.	30.0	80.5	15.7	57.1	
32	Euphorbiaceae	Leaves	*Manihot esculentus* L.	30.4	106	37.1	95.8	
33	Nyctaginaceae	Leaves	*Boerhavia diffusa* L.	30.4	52.7	7.3	14.4	
34	Asclepiadaceae	Leaves	*Calotropis procera* (Aiton) W.T.Aiton	31.1	77.4	11.2	53.7	
35	Portulacaceae	Leaves	*Talinum triangulare* (Jacq.) Willd.	32.1	128.2	20.4	57.9	
36	Olacaceae	Leaves	*Olax subscorpioidea* Oliv.	32.4	66.4	22.1	41.6	
37	Sapotaceae	Leaves	*Synsepalum dulcificum* (Schumach. & Thonn.) Daniell	33.0	53.9	12.9	25.7	
38	Cyperaceae	Leaves	*Cyperus esculentus* L.	33.0	103.0	23.0	74.0	
39	Amaranthaceae	Leaves	*Amaranthus spinosus* L.	33.6	42.7	17.5	41.1	
40	Verbenaceae	Leaves	*Duranta erecta* L	33.7	62.9	8.8	22.0	
41	Sapindaceae	Leaves	*Paullina pinnata* L.	33.8	70.4	22.0	67.2	
42	Boraginaceae	Leaves	*Heliotropium indicum* L.	34.1	104.9	45.2	116	
43	Capparaceae	Leaves	*Capparis fascicularis* DC.	34.6	73.2	16.7	43.9	
44	Tiliaceae	Leaves	*Grewia carpinifolia* Juss.	35.0	92.2	11.6	50.8	
45	Passifloraceae	Leaves	*Adenia cissampeloides* (Planch. ex Benth.) Harms	35.2	103.3	12.0	91.2	

Table S1. continued.

No. of Plants	Family	Part used	Scientific Names	Growth Elongation (% of Control)				Criteria
				10 mg		50 mg		
				Radicle	Hypocotyl	Radicle	Hypocotyl	
**46**	Fabaceae	Leaves	*Tetrapleura tetraptera* Taub.	35.7	69.2	36.4	67.3	
**47**	Sapindaceae	Roots	*Paullinia pinnata* L.	36.1	92.7	20.6	95.7	
**48**	Cyperaceae	Leaves	*Cyperus rotundus* L.	36.3	54.7	21.9	43.8	
**49**	Fabaceae	Leaves	*Baphia nitida* Lodd.	36.1	84.3	13.1	47.5	
**50**	Fabaceae	Leaves	*Senna alata* (L).Roxb.	37.1	77.4	13.2	30.9	
			M	25.4	63.5	14.2	42.3	
			SD	9.3	26.8	8.6	24.9	
		****	M-2.0SD	6.8				
		***	M-1.5SD	11.5				
		**	M-1.0SD	16.1				
		*	M-0.5SD	20.7				

[1] Evaluation of allelopathic potential was done using the Sandwich method (Fujii et al., 2002). The sandwich bioassay is used to assess the potential plant growth inhibitory activity of plants that are exhibited through leachates.[2] M-Average, SD- Standard deviation. [3] Criteria: Stronger inhibitory activity on the radicle with increasing number of *. * M—0.5 (SD), ** M—1(SD), *** M—1.5(SD), and **** M—2.5(SD). .
